# Quality and antioxidant activity of dandelion root infusions as affected by cold plasma pretreatment

**DOI:** 10.1002/fsn3.3791

**Published:** 2023-10-30

**Authors:** Berfin Eda Elcik, Celale Kirkin

**Affiliations:** ^1^ Department of Food Engineering, Faculty of Chemical and Metallurgical Engineering Istanbul Technical University Istanbul Türkiye

**Keywords:** antioxidant activity, cold plasma, color, dandelion root, infusions

## Abstract

Ground and unground dandelion roots were subjected to dielectric barrier discharge cold plasma (DBDCP) at 40 kV for 0 (control), 10, or 20 min. Then, infusions of the pretreated dandelion roots in water were prepared, and the changes in color, total phenolic content (TPC), antioxidant activity, and sensory properties were investigated. The 20‐min pretreatment increased the b* value, TPC, antioxidant activity, and sage odor of the ground dandelion root infusions compared with the control, whereas decreases in the TPC, antioxidant activity, and sage odor were noted in the 10‐min pretreated infusions of the unground roots. DBDCP pretreatment did not affect the overall likeliness of infusions of ground and unground roots. In addition, the TPC, antioxidant activity, and overall likeliness of infusions of the ground dandelion roots were higher than those of the unground samples. In conclusion, it can be said that the DBDCP pretreatment can be utilized to improve the TPC and antioxidant activity of ground dandelion roots.

## Graphical abstract

Dandelion root samples were subjected to dielectric barrier discharge cold plasma, and the infusions in water were prepared. The changes in color, total phenolic content, antioxidant activity, and sensory properties were evaluated.

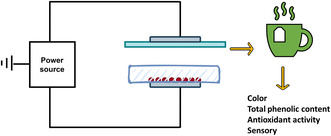

## 1 INTRODUCTION

Dandelion (*Taraxacum officinale*), a perennial herb, has been widely recognized for its extensive medicinal properties and nutritional value (Di Napoli & Zucchetti, 2021; Lis & Olas, 2019; Martinez et al., 2015; Zhu et al., 2017). It grows naturally in the northern hemisphere (Cai et al., 2019; Di Napoli & Zucchetti, 2021; Lis & Olas, 2019; Petkova et al., 2015; You et al., 2010). The root of *Taraxacum officinale*, commonly referred to as the dandelion root, has gained attention due to its health benefits and antioxidant capacity (Choi et al., 2010; González‐Castejón et al., 2012; Radoman et al., 2023). One of the ways of including dandelion root in the diet is through its consumption as tea or coffee, as it is a good source of phenolics, flavonoids, and other bioactive compounds (Abdel‐Moemin & Aboraya, 2014; González‐Castejón et al., 2012; Lis & Olas, 2019). Several potential health effects (such as hepatoprotective, hypolipidemic, and anti‐cancer) of dandelion root and its extracts have been reported previously (Chatterjee et al., 2010; Choi et al., 2010; Ovadje et al., 2016; Pfingstgraf et al., 2021; You et al., 2010).

In recent years, there has been a growing interest in using non‐thermal food processing techniques that can preserve the functional and nutritional attributes of food products better than conventional methods (Tsevdou et al., 2022). Cold plasma is a non‐thermal technology that can be used for the processing of foods at low temperatures. This treatment involves the utilization of charged and highly reactive gaseous species to interact with food components, offering several advantages, such as microbial inactivation and extended shelf life, while preserving the bioactive compounds (Lacombe et al., 2017; Pankaj & Keener, 2017). Other studies have also reported that dandelion supplementation could improve growth, immune response, antioxidant activity, and nutritional capacity in fish (Du et al., 2022; Yu et al., 2022; Zhao et al., 2022) and enhance lactation, antioxidant activity, and serum carbohydrate and amino acid metabolism in cows (Li, Mei, et al., 2023). Similar effects on chickens upon supplementation with dandelion tannins have also been reported (Li, Sun, et al., 2023).

It is possible to use cold plasma to improve the efficiency of various food processes, such as cooking, curing, drying, extraction, and hydrogenation, as reported by de Araújo Bezerra et al. (2023). The use of cold plasma as a pretreatment can improve the extraction of bioactive components, such as anthocyanins, essential oils, flavonoids, galactomannan, and phenolics, from various food products (Bao et al., 2020a, 2020b; Keshavarzi et al., 2020; Li, Li, et al., 2023; Pogorzelska‐Nowicka et al., 2021; Pragna et al., 2019; Rashid et al., 2020). Cold plasma also has the potential to improve the antioxidant activity and phenolic composition of herbal infusions. For instance, cold plasma treatment of brown rice grains improved the phenolic content and antioxidant activity of the corresponding aqueous infusions (Park et al., 2020).

It can be said that the potential enhancement of the quality of herbal infusions upon cold plasma pretreatment can promote the adoption of this technique by the food industry to improve production efficiency. Despite the considerable potential of cold plasma in enhancing food processing efficiency and preserving antioxidant capacity, no research has been conducted on the effects of cold plasma pretreatment on the quality of dandelion root infusions to the best of our knowledge. Thus, this study aimed to evaluate the utilization of cold plasma as a pretreatment in the preparation of dandelion root infusions and its effects on color, phenolic content, antioxidant activity, and sensory properties. It is also intended that the study serves as an example for the industry of the potential of cold plasma technology in food processing.

## 2 MATERIALS AND METHODS

### 2.1 Preparation of the samples

Dandelion (*Taraxacum officinale*) roots were obtained from a local vendor in Izmir. Some roots were ground using a coffee grinder (Bosch). The rest of the roots were kept unground for use in the cold plasma treatment.

### 2.2 Cold plasma treatment

**FIGURE 1:**
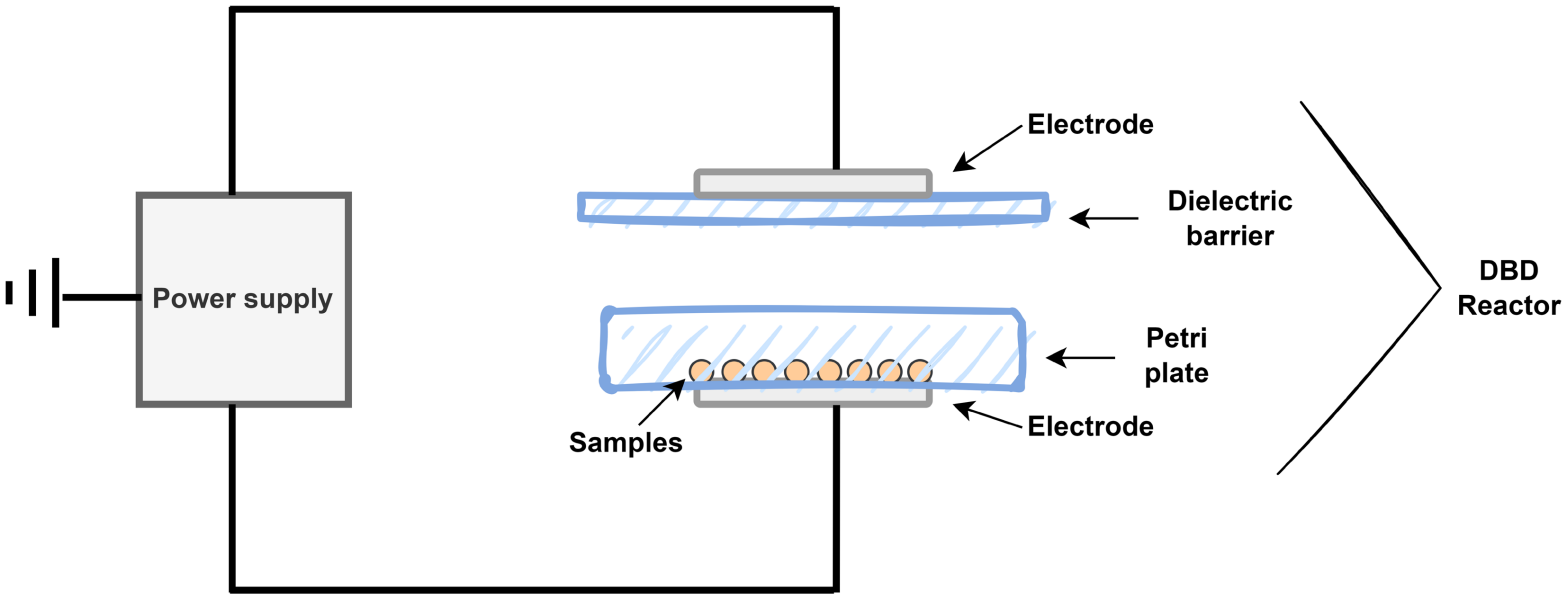
Schematic diagram of the dielectric barrier discharge cold plasma (DBDCP) system.

The dielectric barrier discharge cold plasma (DBDCP) was generated using the experimental setup shown in Figure 1. The system consisted of a high‐voltage (40 kV, 56 kHz, 10 mA) direct current power supply (Asentek) and a dielectric barrier discharge (DBD) reactor. The reactor consisted of two parallel cylindrical stainless‐steel electrodes with a diameter of 120 mm and a thickness of 4 mm. The top electrode was coupled to a glass barrier (140 mm diameter, 2 mm thickness). The samples (3 g) were placed in a glass petri plate (without the lid) with a diameter of 100 mm and a thickness of 1.5 mm, and the petri plate was placed on the ground electrode. The gap between the bottom of the petri plate and the surface of the glass barrier coupled to the top electrodes was 11 mm. Therefore, the plasma discharge was obtained between the electrodes, separated by two dielectric barriers. All samples were subjected to DBDCP for 0, 10, or 20 min. All treatments were repeated three times.

### 2.3 Preparation of the infusions

One gram of each sample was infused in 100 mL of distilled water at 95°C for 4 min. The unground dandelion root samples were placed in hot water in stainless steel sieves, which were removed after the infusion process. On the other hand, the ground samples were added to the water directly, and after 4 min, the infusion was centrifuged for 5 min at 2575 *g*.

### 2.4 Color measurement

The CIE L*, a*, and b* values of the infusions were measured using a chromameter (CR400, Konika‐Minolta). The hue angle (*h*°) and chroma (C*) values of the samples were calculated using Equations (1) and (2), respectively.

(1) $$h^{{}^{\circ}}=\arctan\left(\frac{b^{*}}{a^{*}}\right)$$

(2) $$C^{*}=\sqrt{{\left(a^{*}\right)}^{2}+{\left(b^{*}\right)}^{2}}$$

### 2.5 Total phenolic content

The total phenolic content of the infusions after diluting with distilled water was assessed using the Folin–Ciocalteu method (Viuda‐Martos et al., 2010). The findings are presented in terms of mg gallic acid equivalent (GAE)/L (mg GAE/L).

### 2.6 Antioxidant activity

The antioxidant activity of the dandelion root infusions was assessed using 2,2‐diphenyl‐2‐picrylhydrazyl (DPPH) radical scavenging activity, 2,2′azinobis 3‐ethylbenzothiazoline‐6‐sulfonic acid diammonium salt (ABTS) radical scavenging, and ferric reducing antioxidant power (FRAP) assays (Re et al., 1999; Viuda‐Martos et al., 2010). The results are expressed in terms of mg 6‐hydroxy‐2,5,7,8‐tetramethylchroman‐2‐carboxylic acid equivalents (TE)/L (mg TE/L).

### 2.7 Sensory analysis

Sensory analysis was conducted with 12 panelists, consisting of personnel from the ITU Food Engineering Department. Approximately 3 mL of each sample was served to the panelists in screw‐cap test tubes. The sage odor (1: poor, 7: strong), color (1: very light, 7: very dark), clarity (1: transparent, 7: cloudy), and overall likeliness (1: dislike very much, 7: like very much) of the samples were evaluated using a 7‐point hedonic scale.

### 2.8 Statistical analysis

Data were evaluated by analysis of variance and Tukey's multiple comparison tests. All data are reported as means and standard deviations using statistical software (Minitab 18, Minitab).

## 3 RESULTS AND DISCUSSION

### 3.1 Color

The effects of DBDCP pretreatment on the L*, a*, b*, *h°*, and C* values of the dandelion root infusions are demonstrated in Table 1. The color values of the infusions prepared from the unground samples were not affected by the DBDCP treatment (*p* > .05), except that the *h*° was lower in the 20‐min pretreated infusions than the control (*p* < .05). On the other hand, the 20‐min DBDCP pretreatment decreased the b* value of the infusions of the ground dandelion root compared with the control (*p* < .05).

**TABLE 1:** Effects of DBDCP pretreatment on the color values of the infusions.

Treatment time (min)	L*	a*	b*	*h*°	C*
Unground dandelion root					
0	24.19 ± 1.73^a^	1.90 ± 0.86^a^	14.36 ± 0.40^a^	58.70 ± 0.92^a^	11.18 ± 0.87^a^
10	24.96 ± 0.88^a^	0.87 ± 0.41^a^	11.69 ± 1.60^a^	56.24 ± 2.08^ab^	10.52 ± 0.92^a^
20	22.16 ± 1.77^a^	2.00 ± 0.59^a^	12.17 ± 1.79^a^	53.21 ± 0.63^b^	8.94 ± 0.12^a^
Ground dandelion root					
0	18.30 ± 0.54^a^	5.84 ± 0.38^a^	9.53 ± 0.72^a^	84.36 ± 1.88^a^	14.48 ± 0.49^a^
10	17.77 ± 0.50^a^	5.79 ± 0.31^a^	8.78 ± 0.90^ab^	85.89 ± 2.692^a^	11.72 ± 1.63^a^
20	23.18 ± 5.41^a^	4.58 ± 1.43^a^	5.97 ± 1.01^b^	81.817 ± 4.37^a^	12.33 ± 1.78^a^

*Note*: Data are the mean ± standard deviation of three replications. Values labeled with different letters are statistically different (*p* < .05).

Contrary to the present study, Pogorzelska‐Nowicka et al. (2021) reported an increase in the a* value of cold plasma‐treated (N_2_ plasma jet, 20 kHz, 1 L/min) water extracts of *Taraxacum officinale*. The effect of cold plasma on color values can vary depending on many parameters, including the experimental setup, power, and treatment time. It was reported that cold plasma treatment caused changes in the color parameters of dried hyssop leaves with increasing voltage and exposure time (Rezaei et al., 2020).

The infusions prepared from the ground dandelion samples exhibited lower L* and b* values and higher a*, *h*°, and C* values compared with the infusions of the unground samples (*p* < .05).

### 3.2 Total phenolic content

The phenolic content of the samples as affected by the DBDCP pretreatment is shown in Table 2. The 10‐min DBDCP pretreatment caused a decrease in the TPC of the infusions of the unground dandelion root compared with the control (*p* < .05), whereas the decrease was lost after the 20‐min treatment (*p* > .05). In contrast, the TPC of the infusions of the ground dandelion root was higher in 20‐min DBDCP pretreated samples than in the control and 10‐min‐pretreated samples (*p* < .05).

**TABLE 2:** Effects of DBDCP pretreatment on TPC (mg GAE/L) of the infusions.

Treatment time (min)	TPC
Unground dandelion root	
0	44.53 ± 9.80^a^
10	27.17 ± 1.52^b^
20	36.28 ± 2.10^ab^
Ground dandelion root	
0	122.12 ± 0.54^b^
10	127.48 ± 3.50^b^
20	140.12 ± 5.16^a^

*Note*: Data are the mean ± standard deviation of three replications. Values labeled with different letters are statistically different (*p* < .05).

It was also reported that plasma treatment for 8 min increased the total polyphenolic content of the water extracts of dandelion (Pogorzelska‐Nowicka et al., 2021). The increase in the total phenolic content of the ground dandelion root infusions can be associated with the enhancement of extraction efficiency by plasma treatment, as suggested by several researchers (Bao et al., 2020a, 2020b; de Araújo Bezerra et al., 2023; Mehta et al., 2022). Moreover, Kenny et al. (2015) stated that 1,5‐dicaffeoylquinic acid, a chlorogenic acid, was the major phenolic compound found in dandelion roots. Also, Schütz et al. (2006) reported that dandelion roots were rich in hydroxycinnamic acids. In addition, several other studies have reported that cold plasma treatment of food products could increase the concentration of these compounds (Garofulić et al., 2015; Herceg et al., 2016; Mehta et al., 2019; Rana et al., 2020). However, the effect of cold plasma on the extraction yield depends on factors such as the food product, exposure time, gas type, power, voltage, and frequency (Bao et al., 2020a; Keshavarzi et al., 2020; Kumar et al., 2023; Munekata et al., 2020; Saremnezhad et al., 2021; Zhang et al., 2022). It is also possible that the extraction of phenolics can be reduced by cold plasma because of the reactions with the plasma‐generated reactive substances (Fernandes & Rodrigues, 2021; Munekata et al., 2020; Pankaj et al., 2017). In addition, some researchers have reported that the phenolic concentration of several food products is better preserved by cold plasma treatment than by conventional methods such as heat treatment, extraction, or alkalization of cocoa (Ahmadian et al., 2023; Mehta et al., 2019; Palabiyik et al., 2023).

The total phenolic content was also affected by particle size, and it was higher in the infusions of the ground dandelion root samples than in the unground samples (*p* < .05). It was observed in another study that the total phenolic content of black tea infusion was higher when the particle size was lower (Salman et al., 2019). Similar findings have also been reported by other researchers (Castiglioni et al., 2015; Lee et al., 2014; Riehle et al., 2014).

### 3.3 Antioxidant activity

The antioxidant activity of the samples as affected by DBDCP pretreatment is demonstrated in Table 3. For the infusions of the unground dandelion root samples, the DBDCP pretreatment did not affect the DPPH‐based antioxidant activity (*p* > .05), but the 10‐min pretreatment decreased the ABTS and FRAP assay‐based antioxidant activity compared with the control (*p* < .05). However, the DPPH, ABTS, and FRAP assay‐based antioxidant activity of the infusions of the ground samples was increased after 20 min of pretreatment compared with the control (*p* < .05). The DPPH radical scavenging activity of the infusions prepared from the 20‐min treated ground dandelion root samples was also higher than that of the 10‐min‐pretreated infusions (*p* < .05).

**TABLE 3:** Effects of DBDCP pretreatment on antioxidant activity (mg TE/L) of the infusions as assessed by DPPH scavenging activity, ABTS radical scavenging, and FRAP assays.

Treatment time (min)	DPPH	ABTS	FRAP
Unground dandelion root			
0	290.06 ± 38.87^a^	49.32 ± 4.37^a^	143.95 ± 13.12^a^
10	217.93 ± 22.55^a^	35.63 ± 0.32^b^	90.17 ± 13.02^b^
20	241.22 ± 44.02^a^	44.45 ± 6.43^ab^	118.29 ± 17.19^ab^
Ground dandelion root			
0	667.84 ± 4.27^b^	118.74 ± 4.27^b^	358.08 ± 5.91^b^
10	694.52 ± 13.04^b^	123.88 ± 6.31^ab^	373.55 ± 1.92^ab^
20	773.66 ± 20.00^a^	134.52 ± 3.34^a^	386.72 ± 13.02^a^

*Note*: Data are the mean ± standard deviation of three replications. Values labeled with different letters are statistically different (*p* < .05).

Similarly, an increase in the antioxidant activity of the cold plasma‐treated water extracts of nine herbs, including *T. officinale*, was also noted by Pogorzelska‐Nowicka et al. (2021). The DBDCP treatment (5 W for 5 min or 15 W for 15 min) under air decreased the antioxidant activity of the water extracts of green tea leaves compared with the control (untreated) as assessed by the DPPH scavenging assay; however, an approximately 41% increase in the antioxidant activity was noted upon the 15 min (15 W) treatment under nitrogen (Keshavarzi et al., 2020). In addition, Park et al. (2020) observed increases in the antioxidant activity of the infusions of brown rice grains with increasing cold plasma treatment time, as assessed by the DPPH and ABTS scavenging methods. On the other hand, Hemmati et al. (2021) reported that the FRAP values and DPPH scavenging activity of green tea powder followed a decreasing pattern with increasing cold plasma voltage and treatment time.

The antioxidant activity of the infusions prepared from the ground samples was higher than that of the unground samples, as assessed by the three methods (*p* < .05). Similarly, several studies have reported that particle reduction improves the extraction of antioxidant components in infusions (Castiglioni et al., 2015; Gião et al., 2009; Goh et al., 2003; Lee et al., 2014; Zhang et al., 2020).

### 3.4 Sensory analysis

The sensory properties of the samples affected by the DBDCP pretreatment are shown in Table 4. The sage odor of the infusions prepared by the ground and unground dandelion root samples was increased by the 20‐min pretreatment compared with that of the 10‐min pretreated and control samples, respectively (*p* < .05). The 10‐min pretreated infusions of the unground dandelion root samples were darker than the control (*p* < .05), whereas the 20‐min pretreated infusions of the ground samples were lighter than the unpretreated (*p* < .05). In addition, the 10‐min pretreated infusions of the unground dandelion root infusions were more transparent than the control and the 20‐min pretreated samples (*p* < .05), and no change in the clarity of the infusions of the ground samples was noted (*p* > .05). Cold plasma treatment did not cause a significant change in the overall likeliness of the infusions (*p* > .05).

**TABLE 4:** Effects of DBDCP pretreatment on sensory properties of the infusions.

Treatment time (min)	Sage odor	Color	Clarity	Overall likeliness
Unground dandelion root				
0	2.03 ± 1.40^ab^	5.00 ± 1.32^b^	2.24 ± 1.12^a^	3.15 ± 1.46^a^
10	1.91 ± 1.23^b^	5.55 ± 1.64^a^	1.85 ± 1.12^b^	3.06 ± 1.30^a^
20	2.61 ± 1.80^a^	5.21 ± 1.36^ab^	2.27 ± 1.21^a^	3.24 ± 1.25^a^
Ground dandelion root				
0	2.42 ± 1.64^b^	3.55 ± 0.88^a^	2.67 ± 1.71^a^	3.69 ± 1.30^a^
10	2.14 ± 1.18^b^	3.42 ± 1.00^ab^	2.56 ± 1.42^a^	3.94 ± 1.19^a^
20	2.92 ± 1.59^a^	3.19 ± 0.86^b^	2.56 ± 1.44^a^	3.94 ± 1.19^a^

*Note*: Data are the mean ± standard deviation of three replications. Values labeled with different letters are statistically different (*p* < .05).

Similarly, Park et al. (2020) stated that cold plasma treatment did not cause a negative effect on the sensory properties of germinated and ungerminated brown rice grains. Moreover, several studies have reported positive (Abdel‐Naeem et al., 2022; Chen et al., 2019), negative (Lee et al., 2012; Olatunde et al., 2020; Yong et al., 2015), or neutral (Lee et al., 2018; Ribeiro et al., 2021) effects of cold plasma treatment on the sensory properties of various food products. These effects depend on the factors that are applied during processing, such as voltage, exposure time, plasma source, gas, and the food product (Roshanak et al., 2023; Sruthi et al., 2022).

The grinding of the dandelion root samples enhanced the sensory properties; the infusions of the ground samples demonstrated stronger sage odor, lighter color, and higher overall likeliness scores compared with the infusions of the unground samples (*p* < .05). The infusions prepared from the ground samples were also evaluated as cloudier than those from the unground samples (*p* < .05).

## 4 CONCLUSIONS

The effects of DBDCP pretreatment on the quality properties of dandelion root infusions varied with exposure time and particle size. The DBDCP pretreatment for 20 min improved the TPC and antioxidant activity of the ground dandelion root infusions. The 20‐min pretreated infusions of ground dandelion roots also exhibited a higher b* value, stronger sage odor, and lighter color compared with the control. On the other hand, the 20‐min pretreatment caused a decrease in the *h*° value of the unground samples. In addition, the 10‐min pretreatment decreased the TPC, FRAP, and ABTS scavenging activity of the unground dandelion roots compared with the control. The 10‐min pretreated infusions were also more transparent and had a weaker sage odor. The TPC, antioxidant activity, and overall likeliness were higher in the infusions of ground dandelion roots than in the unground roots. In conclusion, DBDCP pretreatment has the potential to improve the quality and antioxidant properties of the infusions of dandelion root. Further studies can focus on the effects of cold plasma pretreatment on the secondary metabolites and individual components of the infusions.

## AUTHOR CONTRIBUTIONS

**Berfin Eda Elcik:** Formal analysis (lead); investigation (equal); writing – original draft (lead); writing – review and editing (supporting). **Celale Kirkin:** Conceptualization (lead); methodology (lead); project administration (lead); resources (lead); supervision (lead); writing – original draft (supporting); writing – review and editing (lead).

## CONFLICT OF INTEREST STATEMENT

The authors declare that they have no conflicts of interest.

## Data Availability

The data that support the findings of this study are available from the corresponding author upon reasonable request.
